# Germline *ERBB3* mutation in familial non-small-cell lung carcinoma: expanding ErbB’s role in oncogenesis

**DOI:** 10.1093/hmg/ddab172

**Published:** 2021-07-19

**Authors:** Aideen M McInerney-Leo, Hui Yi Chew, Po-Ling Inglis, Paul J Leo, Shannon R Joseph, Caroline L Cooper, Satomi Okano, Tim Hassall, Lisa K Anderson, Rayleen V Bowman, Michael Gattas, Jessica E Harris, Mhairi S Marshall, Janet G Shaw, Lawrie Wheeler, Ian A Yang, Matthew A Brown, Kwun M Fong, Fiona Simpson, Emma L Duncan

**Affiliations:** The University of Queensland Diamantina Institute, The University of Queensland, Woolloongabba, QLD 4102, Australia; The University of Queensland Diamantina Institute, The University of Queensland, Woolloongabba, QLD 4102, Australia; Cancer Care Services, Royal Brisbane and Women’s Hospital, Herston QLD 4029, Australia; Australian Translational Genomics Centre, Institute of Health and Biomedical Innovation, School of Biomedical Sciences, Queensland University of Technology (QUT), Translational Research Institute, 37 Kent St, Woolloongabba, QLD 4102, Australia; The University of Queensland Diamantina Institute, The University of Queensland, Woolloongabba, QLD 4102, Australia; Department of Anatomical Pathology, Pathology Queensland, Princess Alexandra Hospital, Brisbane, QLD 4102, Australia; University of Queensland Thoracic Research Centre, Faculty of Medicine, The University of Queensland, 288 Herston Road, Herston, QLD 4006, Australia; The University of Queensland Diamantina Institute, The University of Queensland, Woolloongabba, QLD 4102, Australia; Centre for Children and Adolescent Brain Cancer, Oncology Department, Queensland Children’s Hospital, South Brisbane, QLD 4101, Australia; Cancer Care Services, Royal Brisbane and Women’s Hospital, Herston QLD 4029, Australia; University of Queensland Thoracic Research Centre, Faculty of Medicine, The University of Queensland, 288 Herston Road, Herston, QLD 4006, Australia; Department of Thoracic Medicine, The Prince Charles Hospital, Rode Road, Chermside, QLD 4032, Australia; Genetic Health Queensland, Royal Brisbane and Women’s Hospital, Herston, QLD 4029, Australia; Australian Translational Genomics Centre, Institute of Health and Biomedical Innovation, School of Biomedical Sciences, Queensland University of Technology (QUT), Translational Research Institute, 37 Kent St, Woolloongabba, QLD 4102, Australia; Australian Translational Genomics Centre, Institute of Health and Biomedical Innovation, School of Biomedical Sciences, Queensland University of Technology (QUT), Translational Research Institute, 37 Kent St, Woolloongabba, QLD 4102, Australia; University of Queensland Thoracic Research Centre, Faculty of Medicine, The University of Queensland, 288 Herston Road, Herston, QLD 4006, Australia; Department of Thoracic Medicine, The Prince Charles Hospital, Rode Road, Chermside, QLD 4032, Australia; Australian Translational Genomics Centre, Institute of Health and Biomedical Innovation, School of Biomedical Sciences, Queensland University of Technology (QUT), Translational Research Institute, 37 Kent St, Woolloongabba, QLD 4102, Australia; University of Queensland Thoracic Research Centre, Faculty of Medicine, The University of Queensland, 288 Herston Road, Herston, QLD 4006, Australia; Department of Thoracic Medicine, The Prince Charles Hospital, Rode Road, Chermside, QLD 4032, Australia; Australian Translational Genomics Centre, Institute of Health and Biomedical Innovation, School of Biomedical Sciences, Queensland University of Technology (QUT), Translational Research Institute, 37 Kent St, Woolloongabba, QLD 4102, Australia; Guy’s and St Thomas’ NHS Foundation Trust and King’s College London NIHR Biomedical Research Centre, King’s College London, SE1 9RT, London, UK; University of Queensland Thoracic Research Centre, Faculty of Medicine, The University of Queensland, 288 Herston Road, Herston, QLD 4006, Australia; Department of Thoracic Medicine, The Prince Charles Hospital, Rode Road, Chermside, QLD 4032, Australia; The University of Queensland Diamantina Institute, The University of Queensland, Woolloongabba, QLD 4102, Australia; Australian Translational Genomics Centre, Institute of Health and Biomedical Innovation, School of Biomedical Sciences, Queensland University of Technology (QUT), Translational Research Institute, 37 Kent St, Woolloongabba, QLD 4102, Australia; Department of Twin Research and Genetic Epidemiology, Faculty of Life Sciences and Medicine, King’s College London, WC2R 2LS, UK

## Abstract

Lung cancer is the commonest cause of cancer deaths worldwide. Although strongly associated with smoking, predisposition to lung cancer is also heritable, with multiple common risk variants identified. Rarely, dominantly inherited non-small-cell lung cancer (NSCLC) has been reported due to somatic mutations in *EGFR/ErbB1* and *ERBB2*. Germline exome sequencing was performed in a multi-generation family with autosomal dominant NSCLC, including an affected child. Tumour samples were also sequenced. Full-length wild-type (wtErbB3) and mutant ERBB3 (mutErbB3) constructs were transfected into HeLa cells. Protein expression, stability, and subcellular localization were assessed, and cellular proliferation, pAkt/Akt and pERK levels determined. A novel germline variant in *ERBB3* (c.1946 T > G: p.Iso649Arg), coding for receptor tyrosine-protein kinase erbB-3 (ErbB3), was identified, with appropriate segregation. There was no loss-of-heterozygosity in tumour samples. Both wtErbB3 and mutErbB3 were stably expressed. MutErbB3-transfected cells demonstrated an increased ratio of the 80 kDa form (which enhances proliferation) compared with the full-length (180 kDa) form. MutErbB3 and wtErbB3 had similar punctate cytoplasmic localization pre- and post-epidermal growth factor stimulation; however, epidermal growth factor receptor (EGFR) levels decreased faster post-stimulation in mutErbB3-transfected cells, suggesting more rapid processing of the mutErbB3/EGFR heterodimer. Cellular proliferation was increased in mutErbB3-transfected cells compared with wtErbB3 transfection. MutErbB3-transfected cells also showed decreased pAkt/tAkt ratios and increased pERK/tERK 30 min post-stimulation compared with wtErbB3 transfection, demonstrating altered signalling pathway activation. Cumulatively, these results support this mutation as tumorogenic. This is the first reported family with a germline *ERBB3* mutation causing heritable NSCLC, furthering understanding of the ErbB family pathway in oncogenesis.

## Introduction

Lung cancer is the leading cause of cancer deaths worldwide (World Health Organisation) (1), with over 80% of cases attributable to smoking. However, lung cancer is also heritable, with heritability of ~ 18% (2). Genome-wide association studies (GWAS) have identified multiple susceptibility loci for lung cancer overall (reviewed (3)), for non-small-cell lung cancer (NSCLC) (4) and for histology-specific subtypes of NSCLC (5) (with specific GWAS in squamous cell carcinoma (6) and adenocarcinoma (7), but not large cell to date). There have also been many reports of familial aggregation of lung cancer, [summarized in (8)], with increased familial risk particularly observed in cases with younger age of onset (9,10), of female gender, and with adenocarcinoma (11), even after adjusting for smoking status (11,12). Linkage and association studies in familial lung cancer have identified unique susceptibility loci as well as confirming loci associated with NSCLC overall and with specific NSCLC subtypes (13–17). Additionally, GWAS have identified unique susceptibility loci for NSCLC cases carrying somatic epidermal growth factor receptor (EGFR) mutations (18,19).

Somatic gain-of-function mutations affecting the tyrosine kinase (TK) domain of EGFR are common in NSCLC, particularly adenocarcinoma, and predict responsiveness to EGFR-targeting tyrosine kinase inhibitors (TKIs) (20). Extremely rarely, germline carriage of *EGFR* mutations has been described in families with autosomal dominant NSCLC, occasionally with additional somatic *EGFR* mutations (21,22). EGFR [ErbB1, Human EGF Receptor (HER) 1] belongs to the ErbB family of receptor TKs, which includes ErbB2 (neu, HER2), ErbB3 (HER3) and ErbB4 (HER4). A germline *ERBB2* mutation was identified in another family with autosomal dominant NSCLC, without additional *ERBB2* somatic variant(s) (23). No paediatric NSCLC were reported in these families; indeed, primary lung cancers in children are very rare (24,25). Notably, none of the loci associated with lung cancer in the many GWAS to date include *EGFR* or other *ERBB* family members (3). The germline mutational profile of lung cancer was recently reviewed: few other genes have been reported with germline mutations in individuals with lung cancer (26).

Here, we report a new causative gene in a family with autosomal dominant NSCLC.

## Results

### Clinical details

**Figure 1:**
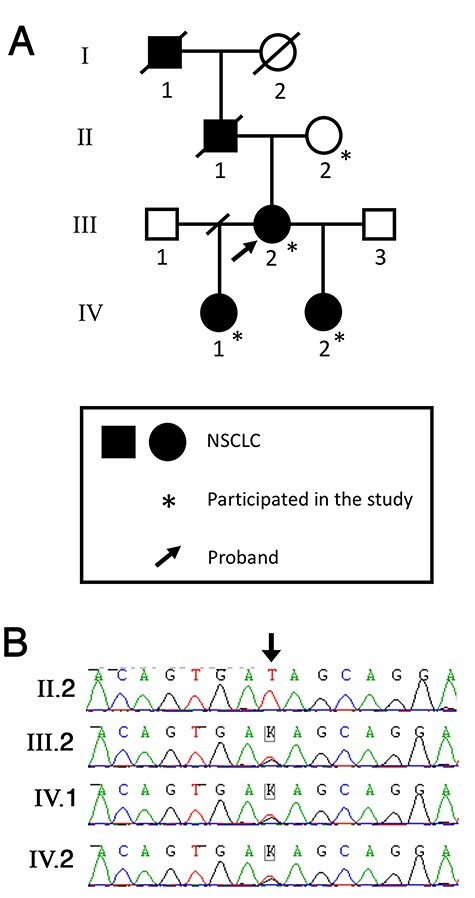
Germline *ERBB3* mutation segregating with NSCLC in an affected family. (**A**). Family Pedigree. (**B**). Sanger sequencing chromatograms of germline DNA, demonstrating heterozygosity for *ERBB3* c.1946 T > G variant (arrow) in three affected individuals and wild type in the proband’s unaffected mother.

The proband (III.2) presented with lung adenocarcinoma aged 51 years. Her father and paternal grandfather, died of NSCLC aged 39 and 34 years, respectively. Two of her five children have lung adenocarcinoma, presenting aged 12 (IV.2) and 30 years (IV.1) (Fig. 1). The proband, her father and grandfather had all smoked at some stage; however, neither child had ever smoked.

### Exome sequencing

Four novel good-quality variants affecting highly conserved bases and with appropriate familial segregation were identified, three of which were predicted damaging by at least two protein prediction algorithms (Table 1; filtering steps presented in Supplementary Material, Table S1). Of these, the *ERBB3* variant (NM_001982; c.1946 T>G; p.Ile649Arg) was of particular interest given the known oncogenic role of ErbB3 itself (27) and of other ErbB family members in heritable NSCLC. In considering the other two variants: *SORBS1* (*Sorbin and SH3 Domain Containing 1*) is involved in cell adhesion, growth factor signalling and cytoskeleton formation but appears mainly to regulate insulin-mediated glucose uptake (28). Although SORBS1 knockdown enhanced MEK/ERK activation and cell migration (29), SORBS1 has not been implicated as a driver in carcinogenesis. *ATG2B* (*Autophagy-Related Protein 2 Homolog B*) is involved in autophagy, a key pathway mediating stress-induced adaptation and cellular damage control, which is exploited by cancer cells to survive starvation and hypoxia (30). Induction of autophagy can occur after chemotherapy, enabling malignant cells to escape and survive its effects; and inhibitors of autophagy can enhance treatment efficacy (31). In NSCLC cell lines, inhibition of autophagy decreased cellular proliferation (32). However, the consensus of opinion regarding autophagy in cancer biology relates more to progression and resistance rather than initiation (33). Thus, although the *ATG2B* variant might influence tumour cell survival and/or chemotherapy responsiveness, it is unlikely to be an oncogenic driver.

**Table 1:** Characteristics of variants fulfilling filtering criteria

Gene	Variant	GERP score	SIFT	MutationTaster	PolyPhen2
*ERBB3* (*Erb-B2 Receptor Tyrosine Kinase 3*)	NM_001982: c.1946 T > G: p.Ile649Arg	3.87	0.00 (deleterious)	0.995 (disease-causing)	0.121 (benign)
*ATG2B (Autophagy related 2B)*	NM_018036: c.4057 T > A: p.Cys1353Ser	5.48	0.05 (deleterious)	0.999 (disease-causing)	0.978 (probably damaging)
*SORBS1 (Sorbin and SH3 domain-containing protein 1)*	NM_001034954: c.2464A > G: p.Ile822Val	6.07	0.05 (deleterious ^*^)	0.999 (deleterious)	0.903 (possibly damaging)

Filtering the data with a less stringent minor allele frequency (MAF) threshold (MAF < 0.001) identified variants in eight additional candidate genes (Supplementary Material, Table S2), of which one (*PAXIP1*) is a genome stability gene previously associated with cancer (34). Somatic copy number variation (CNV) of *PAXIP1* has also been associated with breast cancer prognosis (35). However, the identified variant (rs199937188) is predicted benign and tolerated by Polyphen (36) and SIFT (37) respectively.

The data were also interrogated for coding variants in genes previously implicated in familial lung cancer (including *EGFR, ERBB2, TP53* and *PARK2*) (26). Seventeen variants were identified, but the only two good-quality coding variants failed to segregate appropriately. Filtering for variants in known DNA damage repair (DDR) genes (Supplementary Material) revealed a single coding variant in *REV3L (*c.1675A>G: p.Iso559Val) in IV.2, with MAF 0.00049 (GnomAD), predicted likely benign in ClinVar; this was thought unlikely to be of clinical significance.

### Tumour sequencing

Sanger sequencing of tumour DNA excluded homozygosity of the *ERBB3* variant (data not shown). Unfortunately, tumour DNA from formalin-fixed paraffin-embedded (FFPE) samples was too degraded for massively parallel sequencing, precluding comprehensive assessment of *ERBB3* somatic variants.

### Immunohistochemistry for ERBB3

ErbB3 is typically upregulated in NSCLC, staining both membrane and cytoplasm (38). Tumour tissue from the proband (III.2) showed weak cytoplasmic ErbB3, with absent staining of normal surrounding lung tissue (Supplementary Material, Fig. S1). Results from other tumour samples were inconsistent; notably, less tissue was made available from these other individuals for this study, as their tumour samples were required to inform their ongoing clinical care.

### ErbB3 expression, folding and cytoplasmic localization

**Figure 2:**
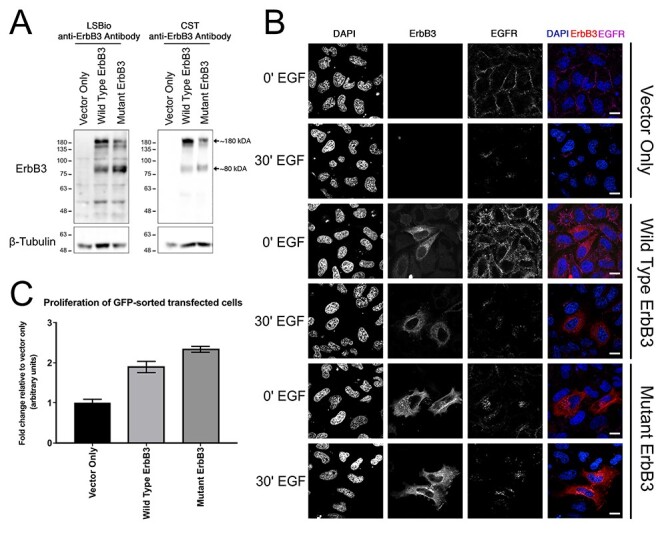
ErbB3 expression, folding, response to stimulation with EGF, and effect on cellular proliferation in HeLa cells transfected with vector-only, wtErbB3, or mutErbB3. (**A**). Western blot of lysates from transfected cells using two different commercial anti-ErbB3 antibodies. MutErbB3 is stably expressed and normally folded. A higher ratio of 80 kDa to full length 180 kDa form (arrows) is observed with mutErbB3 compared with wtErbB3. (**B**). Transfected HeLa cells fixed pre- (0’) and post (30’)-EGF stimulation and immunostained for ErbB3 (red), endogenous EGFR (purple) and nuclei stained using DAPI (blue). Right column shows merged image. Scale bars, 20 μm. Without stimulation EGFR co-localized with mutErbB3 in concentrated puncta, less evident with wtErbB3. After stimulation, both wtErbB3 and mutErbB3 increased in the perinuclear region, co-localizing with EGFR. (**C**)**.** Proliferation assay of transfected cells quantified and described as fold change relative to vector only (data shown as mean ± standard error of mean). MutErbB3-transfected cells showed increased rates of proliferation.

Both wtErbB3 and mutErbB3 were folded and expressed stably (Fig. 2A) with similar subcellular localization (Fig. 2B). Cytoplasmic organelle distribution mirrored that of endogenous ErbB3 (in other, non-HeLa cells; data not shown). Compared with wtErbB3, cells expressing mutErbB3 showed a higher ratio of the 80 kDa to full-length (~180 kDa) forms (Fig. 2A). Without epidermal growth factor (EGF) stimulation, EGFR co-localized with mutErbB3 in concentrated puncta in the endosomal system, which was less evident with wtErbB3 (Fig. 2B). After EGF stimulation, both wtErbB3 and mutErbB3 increased in the perinuclear region (Fig. 2B), also co-localizing with EGFR at this time-point (Fig. 2B, 30′ time-point).

### Cell proliferation

HeLa cells expressing mutErbB3 demonstrated increased cellular proliferation, when compared with HeLa cells expressing either wtErbB3 or vector only (Fig. 2C).

### Signalling pathway activation

**Figure 3:**
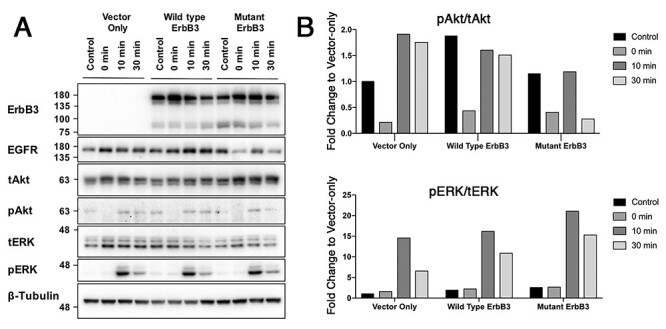
Analysis of protein expression and downstream signalling pathway activation in HeLa cells transfected with vector-only, wtErbB3 or mutErbB3 constructs, before starvation (control), after 3-h starvation (0 min) followed by EGF stimulation (assessed at 10 min and 30 min). (**A**). Western blots of ErbB3, EGFR, phospho-Akt (pAkt), total Akt (tAkt), phospho-ERK (pERK) and total ERK (tERK), performed on transfected cell lysates. Following starvation and EGF stimulation, mutErbB3-transfected cells demonstrated decreased EGFR levels compared with wtErbB3. (**B**). Ratios of pAkt/tAkt (upper graphs), and pERK/tERK (lower graphs) quantified and normalized to β-tubulin. By 30 min, mutErbB3-transfected cells show decreased pAkt/tAkt ratio and increased pERK/tERK ratio compared with wtErbB3-transfected cells. Both blot images and ratio quantification are representative of at least three separate biological replicates.

After EGF stimulation, expression levels of mutErbB3 and wtErbB3 were comparable at all time-points, though, EGFR levels decreased over time in cells expressing mutErbB3 compared with wtErbB3 (Fig. 3A).

Cells expressing either mutErbB3 or wtErbB3 had increased pAkt levels, compared with vector-only transfected cells (Fig. 3B). After starvation followed by 10-min EGF stimulation, similar pAkt/tAkt ratios were observed in mutErbB3, wtErbB3, and vector-only transfected cells (Fig. 3B). However, by 30 min, mutErbB3-transfected cells had decreased pAkt/tAkt ratios compared with wtErBB3 and with vector-only transfected cells (Fig. 3B). Of note, mutErbB3-expressing cells had increased pERK at the 30-min time-point (Fig. 3B). These findings show that mutErbB3 is changing the signalling activation profile in response to ligand stimulation.

Together, these results suggest that the EGFR/mutErbB3 heterodimer is more efficiently activated, internalized and degraded, compared with EGFR/wtErbB3.

## Discussion

We have identified a novel germline mutation in *ERBB3* segregating with autosomal dominant NSCLC. We demonstrate that mutErbB3 is stably expressed, functional with EGFR heterodimerization and signalling, with an increased ratio of 80 kDa versus full-length 180 kDa ErbB, a faster time-course of signalling activation and degradation, and increased cellular proliferation, compared with wtErbB3. These results support this mutation as the oncogenic driver of NSCLC in this family.

Multiple studies demonstrate the importance of ErbB3 in oncogenesis generally and NSCLC specifically (39). *ERBB3* is part of a five-gene expression ‘signature’ predictive of relapse-free and overall survival in NSCLC, independent of age, gender, stage and histological characteristics (40). In a gene expression study of 10 ‘signature’ genes in early lung adenocarcinoma, a two-gene signature, comprising only *ERBB3* and *BRCA1* expression was an independent risk factor in predicting survival, improving the discriminatory power of conventional classification systems (41). Other studies also identified increased ErbB3 expression correlating with shorter survival in NSCLC (42). Within NSCLC, *ERBB3* expression is higher in adenocarcinoma compared with squamous (43) and other forms of lung cancer (44); and circulating *ERBB3* mRNA levels correlate with higher TNM stage and poorer survival in adenocarcinoma (45).

The mutation reported here (c.1946 T > G; p.Ile649Arg) lies in a conserved transmembrane motif, key to dimerization (46). Notably the ERBB2 variant (p.Gly660Asp) previously associated with autosomal dominant NSCLC is located in the corresponding transmembrane motif of ERBB2/HER2 (23). Although germline *ERBB3* variants have been reported previously (47), pathogenic variants have been reported extremely rarely—viz., a germline *ERBB3* mutation (c.4009G > A;p.Ala1337Thr), affecting the C-terminus of the protein, was reported in association with familial erythroleukemia (48), and a homozygous loss-of-function mutation in *ERBB3* was associated with lethal congenital contractural syndrome type 2 (OMIM 607598) in two Israeli-Bedouin families (49). ClinVar (50) contains 40 *ERBB3* variants, of which two are categorized pathogenic and 13 likely pathogenic. Both pathogenic variants and three of the likely pathogenic variants are associated with congenital contracture syndrome; one of the likely pathogenic variants is associated with familial erythroleukemia (as above). The nine remaining likely pathogenic variants are reported in association with malignancy, identified through tumour sequencing in two large cohorts (27,51). As paired germline samples were not sequenced, it is unknown whether these variants were germline or somatic. None of the variants were reported in association with lung carcinoma and none are proximal to the mutation reported in our case report.

Although it has been hypothesized that germline polymorphisms in ErbB genes would contribute to lung cancer risk (52), no such associations have been identified in GWAS of lung cancer to date (neither lung cancer overall nor individual histopathological subtype) (4–7,13,53). Indeed, *ERBB3* has been ‘relatively underinvestigated’ in lung cancer (52). A very small single-candidate gene study suggested association of a variant in the *ERBB3* promoter region with lung cancer—but only with analysis restricted to a recessive model and limited to a non-smoking subset of 119 cases and 191 controls (*P* = 0.037) (54). Reduced ERBB3 expression was reported with the protective allele, consistent with an oncogenic role of ERBB3; however, these results have not been replicated in an independent cohort. Reviewing TCGA (https://www.cancer.gov/tcga) and COSMIC (55), somatic *ERBB3* mutations—whilst common in colonic and gastric carcinomas—appear to be rare in NSCLC (Supplementary Material, Table S3). However, a study assessing CNVs in ErbB genes found that half of all lung adenocarcinomas have CNVs of *EGFR, ERBB2, ERBB3* and *ERBB4*, with higher CNV number corresponding to poorer prognosis (56).

Our germline *ERBB3* mutation is novel for NSCLC and has not been reported (either as a somatic or germline mutation) in any other tumour type. Attributing causality to a variant segregating within a relatively small family just because of its rarity can lead to misattribution (57,58); hence, our comprehensive functional assessment supporting this mutation as causative. Unsurprisingly, given the rarity of autosomal dominant NSCLC, no additional families were available for replication. However, our data concord with previous reports of germline mutations in ErbB family members *EGFR* (*ERBB1)* (59) and *ERBB2* (23) in other pedigrees with autosomal dominant NSCLC. Poor-quality tumour DNA precluded assessment of *ERBB3* mutation(s) in our family, noting that somatic mutations were not identified in the single family with the germline *ERBB2* mutation and NSCLC (23) and inconsistently in individuals and families with *EGFR/ERBB1* mutations (59). Although ErbB3 is expressed widely, this family has not manifested other malignancies (the proband has had non-cancerous colonic polyps). The apparent tissue specificity for malignancy is unclear, although again is consistent with NSCLC families with *EGFR* (ERBB1) (59) and *ERBB2* (23) mutations.

Our functional data support the identified variant as tumorogenic. Normally, ErbB3 (180 kDa) is expressed as a transmembrane protein dimerized with another ErbB family member; upon activation, the heterodimer is internalized via ligand-induced receptor-mediated endocytosis to endosomes and subsequently trafficked to lysosomes for proteolytical degradation. Additionally, some transmembrane ErbB3 is directly cleaved, forming a cytoplasmic stable and active 80 kDa form, which effects are normally offset by the tumour suppressor p14ARF sequestering the 80 kDa form for degradation (60). Our results suggest that mutErbB3 is more prone to cleavage, resulting in increased amounts of the cytoplasmic 80 kDa form; moreover, this increase in the 80 kDa form may exceed the sequestration capacity of p14ARF. Further, the 80 kDa form may independently drive proliferation, as it can increase transcription of proliferative genes without requiring activation of cytoplasmic pathways (60). Notably, our results demonstrated increased cellular proliferation in mutErbB3-transfected cells compared with wtErbB3.

We also demonstrate that mutErbB3 co-localizes with EGFR, with EGFR levels decreasing over time in mutErbB3-transfected cells, compared with wtErbB3-transfected cells. Together, these results suggest that EGFR/mutErbB3 dimers internalize and reach the late endosomal/lysosomal system faster than wtErbB3-transfected cells, consistent with more rapid signal transduction with mutErbB3. Further, activation profiles of downstream signalling pathways differed in mutErbB3-transfected cells compared with wtErbB3-transfected cells; these pathways affect transcriptional regulation of cell proliferation and migration, both critical for cancer initiation and metastasis (27).

Lastly, although we demonstrate clear oncogenic consequences of the *ERBB3* mutation and detail our reasoning for focusing on this variant rather than the variants in *ATG2B* and *SORBS1*, an oncogenic contribution from these other variants cannot be completely excluded. We were unable to assess the proband’s as yet clinically unaffected children, because of ethical constraints; moreover, these children were quite young. Thus, further assessment of segregation could not be used to inform our reasoning. The quality and quantity of available tumour samples limited the ability to perform additional functional studies of these other variants (as well as limiting assessment for additional somatic mutations in *ERBB3*, as discussed above).

Our results may have clinical implications beyond genetic counselling. Both germline and somatic EGFR mutations affect NSCLC responsiveness to TKIs, and ErbB family-targeted therapy can induce prolonged progression-free survival specifically in individuals with TK domain mutations (including NSCLC) (61). The identified *ERBB3* mutation does not lie within this domain; thus, ErbB family inhibitors may not benefit this family. However, ErbB3 downregulation (e.g. by siRNA) can restore tumour responsiveness to various therapeutic approaches, including TKIs, potentially of clinical relevance for this family (61).

In conclusion, we report the first family with heritable NSCLC segregating with a germline mutation in *ERBB3*, with functional data strongly supporting this mutation as oncogenic.

## Materials and Methods

This study was approved by The Prince Charles Hospital Metro North Human Research Ethics Committee (approval HREC/13/QPCH/216). Participants gave informed written consent.

Detailed methods are presented in Supplementary material. Briefly, exome sequencing was performed on germline DNA in a multi-generational family with autosomal dominant NSCLC (Fig. 1). Given the rarity of autosomal dominant NSCLC, and paediatric lung malignancies overall (24,25), analysis focussed on rare variants (previously unreported and with MAF < 0.001), assessed against internal and external databases (e.g. gnomAD (47), 1000 Genomes (62) and dbSNP137 (63)). The data were also interrogated to rule out a rare, deleterious variant in 55 DDR genes (see Supplementary Material for gene list) particularly given the extremely early onset of disease in the 12-year, IV.2.

FFPE samples were obtained from individuals III.2, IV.1 and IV.2, with DNA extracted and sequenced. Expression and localization of ErbB3 in normal and tumour tissue was assessed by immunohistochemistry.

Full-length wild-type (wtErbB3) and mutant (mutErbB3, c.1946 T > G: p.Iso649Arg) *ERBB3* expression constructs were produced and transfected into HeLa cells (which do not express endogenous ErbB3 or ErbB2 but do express EGFR (ErbB1), the preferred dimerization partner of ErbB3 (64)). To evaluate protein size and conformation, western blotting was performed on lysates from transfected HeLa cells (vector-only, wtErbB3 or mutErbB3), probed with commercial antibodies against ErbB3 with β-tubulin used as protein-loading control. To assess localization pre- and post-stimulation, transfected cells were either fixed (0’) or stimulated with 10 ng EGF-Alexa Fluor 488 (30’) prior to fixation and immunostained for ErbB3 and endogenous EGFR, with nuclei stained using DAPI. Transfected cells (vector-only, wtErbB3 or mutErbB3, co-transfected with green fluorescence protein) were separated by fluorescence-activated cell sorting and proliferation rate assessed. Signalling pathway activation of ErbB3 and mutErbB3 transfected cells were analyzed by immunoblotting for ErbB3, EGFR, Akt (phospho- and total) and ERK (phospho- and total) in cells grown in full serum (control), 3-h post-serum starvation (0) and post-EGF stimulation (10 ng/ml) at 10 and 30 min. Relative protein expression was quantified and the ratio normalized to β-Tubulin (used as a loading control) to enable quantification of phospho- to total-Akt (pAkt/tAkt) and phospho- to total-ERK (pERK/tERK). Results are presented without formal statistical assessment as is conventional for these analyses (65).

## Supplementary Material

ERBB3_SUPPLEMENTAL_DATA_13_06_21_ddab172
